# Crystal structure of *catena*-poly[bis­[μ_3_-2-(2-nitro­phen­yl)acetato-κ^3^ *O*:*O*:*O*′]disilver(I)]

**DOI:** 10.1107/S2056989015007616

**Published:** 2015-04-25

**Authors:** Muhammad Danish, Muhammad Nawaz Tahir, Sana Iftikhar, Nazir Ahmad

**Affiliations:** a Department of Chemistry, Institute of Natural Sciences, University of Gujrat, Gujrat 50700, Pakistan; b Department of Physics, University of Sargodha, Sargodha, Punjab, Pakistan; c State Key Laboratory of Materials, Synthesis and New Technology, Wuhan University of Technology, Wuhan 430070, China

**Keywords:** crystal structure, silver(I) complex, 2-(2-nitro­phen­yl)acetic acid, hydrogen bonding

## Abstract

The title compound, [Ag_2_(C_8_H_6_NO_4_)_2_]_*n*_, is a silver complex of 2-(2-nitro­phen­yl)acetic acid. The mol­ecules are not conventional crystallographic inversion dimers but consist of two independent ligands and two Ag^I^ ions, each with a distorted T-shaped coordination environment. The dihedral angles between acetate groups and the benzene rings are 51.1 (2) and 57.9 (2)°. The nitro groups are oriented at dihedral angles of 23.6 (5) and 32.3 (3)° with respect to the parent benzene rings. The dimers form polymeric chains along the *a*-axis direction. The Ag⋯Ag separation within a dimer is 2.8200 (5) and between symmetry-related dimers is 3.6182 (5) Å. The polymeric chains are inter­linked by C—H⋯O hydrogen-bond inter­actions.

## Related literature   

For related structures see: Danish *et al.* (2011*a* ▸,*b* ▸, 2015*a* ▸,*b* ▸); Li *et al.* (2011 ▸)
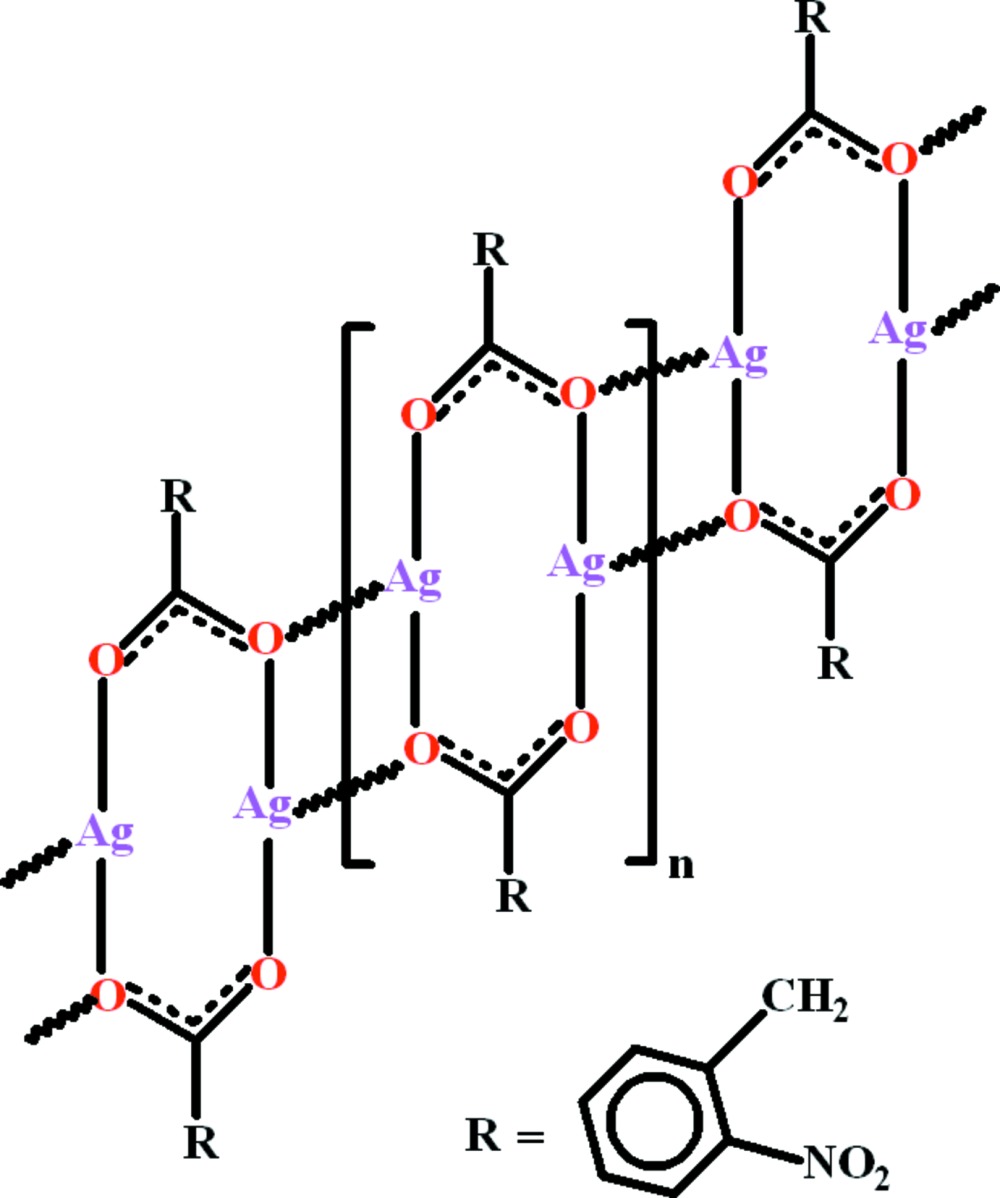


## Experimental   

### Crystal data  

[Ag_2_(C_8_H_6_NO_4_)_2_]
*M* *_r_* = 576.02
Monoclinic, 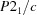

*a* = 5.5249 (3) Å
*b* = 15.8838 (10) Å
*c* = 20.0257 (11) Å
β = 96.853 (3)°
*V* = 1744.83 (17) Å^3^
*Z* = 4
Mo *K*α radiation
μ = 2.30 mm^−1^
*T* = 296 K
0.37 × 0.22 × 0.18 mm

### Data collection  

Bruker Kappa APEXII CCD diffractometer
Absorption correction: multi-scan (*SADABS*; Bruker, 2005 ▸) *T*_min_ = 0.485, *T*_max_ = 0.682
14170 measured reflections
3781 independent reflections
3113 reflections with *I* > 2σ(*I*)
*R*_int_ = 0.027

### Refinement  

*R*[*F* ^2^ > 2σ(*F* ^2^)] = 0.033
*wR*(*F* ^2^) = 0.066
*S* = 1.10
3781 reflections
253 parameters
H-atom parameters constrained
Δρ_max_ = 0.47 e Å^−3^
Δρ_min_ = −0.55 e Å^−3^

### 

Data collection: *APEX2* (Bruker, 2007 ▸); cell refinement: *SAINT* (Bruker, 2007 ▸); data reduction: *SAINT*; program(s) used to solve structure: *SHELXS97* (Sheldrick, 2008 ▸); program(s) used to refine structure: *SHELXL2014* (Sheldrick, 2015 ▸); molecular graphics: *ORTEP-3 for Windows* (Farrugia, 2012 ▸) and *PLATON* (Spek, 2009 ▸); software used to prepare material for publication: *WinGX* (Farrugia, 2012 ▸) and *PLATON*.

## Supplementary Material

Crystal structure: contains datablock(s) global, I. DOI: 10.1107/S2056989015007616/fk2086sup1.cif

Structure factors: contains datablock(s) I. DOI: 10.1107/S2056989015007616/fk2086Isup2.hkl

**Fig. 1.**
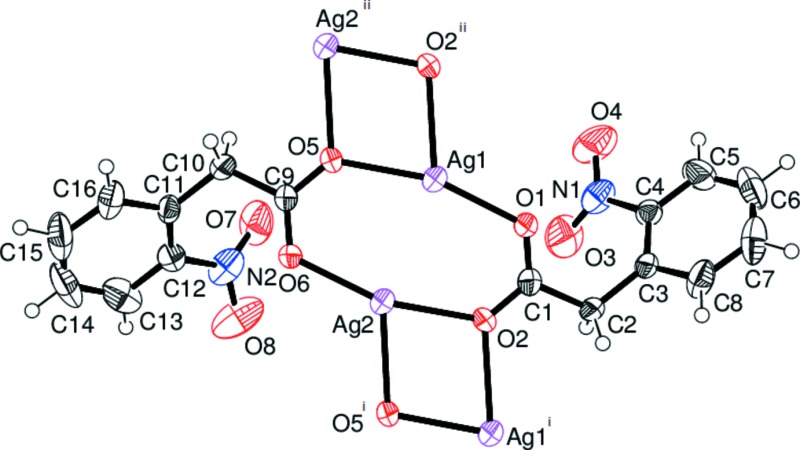
Molecular structure of the title compound. Anisotropic displacement ellipsoids are drawn at the 50% probability level. H-atoms are shown by small circles of arbitrary radii.

. DOI: 10.1107/S2056989015007616/fk2086fig1.tif
Mol­ecular structure of the title compound. Anisotropic displacement ellipsoids are drawn at the 50% probability level. H-atoms are shown by small circles of arbitrary radii.

**Fig. 2.**
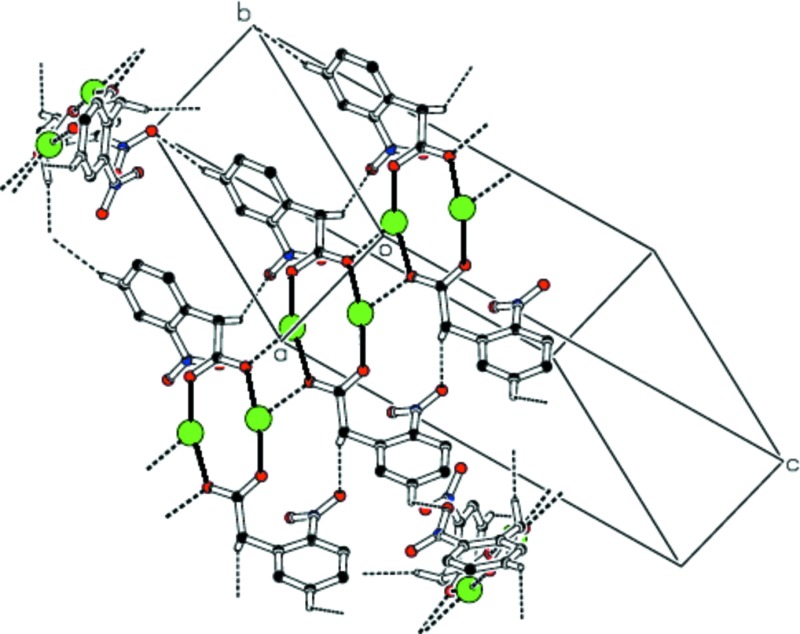
Crystal packing which shows that molecules form polymeric network due to interlinkage of dimers. The dimers are interlinked due to H-bondings. The H-atoms not involved in H-bondings are omitted for clarity.

. DOI: 10.1107/S2056989015007616/fk2086fig2.tif
Crystal packing which shows that mol­ecules form polymeric network due to inter­linkage of dimers. The dimers are inter­linked due to H-bondings. The H-atoms not involved in H-bondings are omitted for clarity.

CCDC reference: 1060272

Additional supporting information:  crystallographic information; 3D view; checkCIF report

## Figures and Tables

**Table 1:** Hydrogen-bond geometry (, )

*D*H*A*	*D*H	H*A*	*D* *A*	*D*H*A*
C2H2*B*O4^i^	0.97	2.53	3.312(6)	138
C10H10*B*O8^ii^	0.97	2.35	3.205(6)	146
C6H6O8^iii^	0.93	2.48	3.322(6)	151
C15H15O7^iv^	0.93	2.59	3.268(6)	130

Symmetry codes: (i) 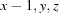; (ii) 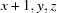; (iii) 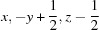; (iv) 
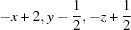
.

## supplementary crystallographic information

### S1. Structural commentary

We have reported the crystal structure of *catena*- poly[[tri­methyl­tin(IV)]-µ-2-(2-nitro­phenyl)­acetato-κ^2^*O*:*O*'] (Danish *et al.*, 2015*a*) and tetra-aqua-bis-[2-(2-nitro­phenyl)­acetato-κ*O*]cobalt(II) (Danish *et al.*, 2015*b*). The title silver complex (I), (Fig. 1) is in continuation of synthesizing various metal complexes with this legand and other studies of these complexes. We have also reported the crystal structures of *catena*-poly[bis-(µ3–2-methyl­benzoato)disilver(I)] (Danish *et al.*, 2011*a*) and *catena*-poly[bis-(µ3–2-methyl-3,5-di­nitro­benzoato)disilver (I)] (Danish *et al.*, 2011*b*) which are related to (I). Similarly, the crystal structures of bis­(*N*,*N*-di­methyl­pyridin-4-amine)-((4-hy­droxy­phenyl) acetato)-silver dihydrate (Li *et al.*, 2011), is related to the title compound.

In (I), the two ligands of (2-nitro­phenyl)­acetatic acid have been coordinated to to two silver ions making a dimer. The structural behaviour of both ligands is different. In one ligand the acetato moiety *A* (O1/C1/C2/O2) and benzene ring *B* (C3–C8) are planar with r.m.s. deviation of 0.0041 and 0.0091 Å, respectively. The dihedral angle between *A*/*B* is 57.87 (17)°. The nitro group is oriented at a dihedtal angle of 23.6 (5)° with the parent benzene ring. In the second ligand the acetato moiety *C* (O5/C9/C10/O6) and benzene ring *D* (C11—C16) are also planar with r. m. s. deviation of 0.0046 and 0.0147 Å, respectively. The dihedral angle between *C*/*D* is 51.13 (16)°. The adjacent nitro group makes dihedral angle of 32.3 (3)° with *D*. The central eight membered ring (Ag1/O1/C1/O2/Ag2/O5/C9/O6) is not exactly planar. The dimers are inter­linked from opposite ends due to Ag—O bonds in the form of one dimensional polymers extending along the *a*-axis. One of the H-atoms of each methlenic group makes H-bonding with adjacent nitro group of parental ligand in the one-dimensional chain. The polymers are inter­linked due to C—H···O inter­actions, where C-atoms are of benzene rings and O-atoms are of nitro groups, therfore, stabilizing the molecules in the form of three dimensional polymeric network ultimately.

### S2. Synthesis and crystallization

The sodium salt of (2-nitro­phenyl)­acetic acid was prepared in water with one molar ratio of (2-nitro­phenyl)­acetic acid and Na(HCO_3_). In this solution one mole of silver nitrate AgNO_3_ (1.08 g) dissolved in water was added and stirred for 5 minutes. Curd white precipitate formed was dissolved by adding few drops of liquid ammonia and kept for crystallization in dark. Needle like colourless crystals were obtained after two weeks.

Yield: 45% Melting Point: 395 K (Decomposes)

### S3. Refinement

Crystal data, data collection and structure refinement details are summarized in Table 1. The H-atoms were positioned geometrically (C–H = 0.93–0.97 Å) and refined riding on the carbon atoms with isotropic displacement parameters U_iso_(H) = 1.2U_eq_(C).

### Crystal data

**Table:**

[Ag_2_(C_8_H_6_NO_4_)_2_]	*F*(000) = 1120
*M**_r_* = 576.02	*D*_x_ = 2.193 Mg m^−^^3^
Monoclinic, *P*2_1_/*c*	Mo *K*α radiation, λ = 0.71073 Å
*a* = 5.5249 (3) Å	Cell parameters from 3113 reflections
*b* = 15.8838 (10) Å	θ = 1.6–27.0°
*c* = 20.0257 (11) Å	µ = 2.30 mm^−^^1^
β = 96.853 (3)°	*T* = 296 K
*V* = 1744.83 (17) Å^3^	Needle, colorless
*Z* = 4	0.37 × 0.22 × 0.18 mm

### Data collection

**Table:**

Bruker Kappa APEXII CCD diffractometer	3781 independent reflections
Radiation source: fine-focus sealed tube	3113 reflections with *I* > 2σ(*I*)
Graphite monochromator	*R*_int_ = 0.027
Detector resolution: 7.80 pixels mm^-1^	θ_max_ = 27.0°, θ_min_ = 1.6°
ω scans	*h* = −7→7
Absorption correction: multi-scan (*SADABS*; Bruker, 2005)	*k* = −20→20
*T*_min_ = 0.485, *T*_max_ = 0.682	*l* = −25→25
14170 measured reflections	

### Refinement

**Table:**

Refinement on *F*^2^	Primary atom site location: structure-invariant direct methods
Least-squares matrix: full	Secondary atom site location: difference Fourier map
*R*[*F*^2^ > 2σ(*F*^2^)] = 0.033	Hydrogen site location: inferred from neighbouring sites
*wR*(*F*^2^) = 0.066	H-atom parameters constrained
*S* = 1.10	*w* = 1/[σ^2^(*F*_o_^2^) + (0.0165*P*)^2^ + 3.0884*P*] where *P* = (*F*_o_^2^ + 2*F*_c_^2^)/3
3781 reflections	(Δ/σ)_max_ < 0.001
253 parameters	Δρ_max_ = 0.47 e Å^−^^3^
0 restraints	Δρ_min_ = −0.55 e Å^−^^3^

### Special details

**Table:**

Geometry. All e.s.d.'s (except the e.s.d. in the dihedral angle between two l.s. planes) are estimated using the full covariance matrix. The cell e.s.d.'s are taken into account individually in the estimation of e.s.d.'s in distances, angles and torsion angles; correlations between e.s.d.'s in cell parameters are only used when they are defined by crystal symmetry. An approximate (isotropic) treatment of cell e.s.d.'s is used for estimating e.s.d.'s involving l.s. planes.
Refinement. Refinement of *F*^2^ against ALL reflections. The weighted *R*-factor *wR* and goodness of fit *S* are based on *F*^2^, conventional *R*-factors *R* are based on *F*, with *F* set to zero for negative *F*^2^. The threshold expression of *F*^2^ > σ(*F*^2^) is used only for calculating *R*-factors(gt) *etc*. and is not relevant to the choice of reflections for refinement. *R*-factors based on *F*^2^ are statistically about twice as large as those based on *F*, and *R*-factors based on ALL data will be even larger.

### Fractional atomic coordinates and isotropic or equivalent isotropic displacement parameters (Å^2^)

**Table:**

	*x*	*y*	*z*	*U*_iso_*/*U*_eq_	
Ag1	0.94971 (5)	0.08333 (2)	0.04106 (2)	0.04649 (10)	
Ag2	0.55095 (5)	−0.00701 (2)	0.08051 (2)	0.04710 (10)	
O1	0.6850 (4)	0.17796 (16)	0.00365 (15)	0.0446 (7)	
O2	0.3776 (5)	0.11268 (17)	0.04293 (16)	0.0507 (8)	
O3	0.6222 (7)	0.3128 (3)	0.10446 (17)	0.0813 (12)	
O4	0.9451 (7)	0.3737 (3)	0.0785 (2)	0.0857 (12)	
O5	1.1302 (5)	−0.02471 (16)	0.09716 (15)	0.0455 (7)	
O6	0.8165 (4)	−0.09755 (16)	0.12586 (14)	0.0422 (7)	
O7	1.0110 (7)	−0.0492 (2)	0.2652 (2)	0.0769 (11)	
O8	0.6646 (7)	−0.0985 (3)	0.28321 (19)	0.0891 (13)	
N1	0.7356 (7)	0.3501 (2)	0.06550 (19)	0.0519 (9)	
N2	0.8701 (7)	−0.1072 (3)	0.26878 (18)	0.0534 (10)	
C1	0.4719 (7)	0.1752 (2)	0.0172 (2)	0.0376 (9)	
C2	0.3050 (7)	0.2495 (2)	0.0012 (2)	0.0439 (10)	
H2A	0.1657	0.2306	−0.0292	0.053*	
H2B	0.2445	0.2672	0.0426	0.053*	
C3	0.4109 (6)	0.3250 (2)	−0.02943 (19)	0.0321 (8)	
C4	0.6114 (7)	0.3702 (2)	−0.00200 (19)	0.0344 (8)	
C5	0.7012 (8)	0.4370 (3)	−0.0343 (3)	0.0583 (12)	
H5	0.8407	0.4645	−0.0149	0.070*	
C6	0.5883 (11)	0.4634 (3)	−0.0944 (3)	0.0666 (14)	
H6	0.6486	0.5095	−0.1158	0.080*	
C7	0.3877 (11)	0.4226 (3)	−0.1231 (2)	0.0654 (14)	
H7	0.3080	0.4410	−0.1640	0.079*	
C8	0.3015 (8)	0.3533 (3)	−0.0914 (2)	0.0531 (11)	
H8	0.1660	0.3248	−0.1122	0.064*	
C9	1.0388 (6)	−0.0834 (2)	0.12825 (19)	0.0338 (8)	
C10	1.2235 (7)	−0.1397 (3)	0.1690 (2)	0.0448 (10)	
H10A	1.3176	−0.1683	0.1379	0.054*	
H10B	1.3352	−0.1038	0.1972	0.054*	
C11	1.1271 (7)	−0.2051 (2)	0.21313 (18)	0.0333 (8)	
C12	0.9564 (7)	−0.1916 (2)	0.25696 (18)	0.0366 (8)	
C13	0.8654 (9)	−0.2561 (3)	0.2933 (2)	0.0609 (13)	
H13	0.7455	−0.2457	0.3211	0.073*	
C14	0.9568 (12)	−0.3357 (3)	0.2873 (3)	0.0778 (18)	
H14	0.8973	−0.3801	0.3108	0.093*	
C15	1.1325 (11)	−0.3498 (3)	0.2474 (3)	0.0747 (17)	
H15	1.1997	−0.4033	0.2453	0.090*	
C16	1.2119 (8)	−0.2866 (3)	0.2102 (2)	0.0518 (11)	
H16	1.3281	−0.2985	0.1815	0.062*	

### Atomic displacement parameters (Å^2^)

**Table:**

	*U*^11^	*U*^22^	*U*^33^	*U*^12^	*U*^13^	*U*^23^
Ag1	0.02941 (16)	0.04213 (18)	0.0682 (2)	0.00658 (13)	0.00686 (14)	0.02080 (15)
Ag2	0.02920 (16)	0.04436 (18)	0.0678 (2)	0.00628 (13)	0.00618 (14)	0.02365 (16)
O1	0.0297 (14)	0.0374 (15)	0.069 (2)	0.0081 (11)	0.0160 (13)	0.0225 (13)
O2	0.0301 (14)	0.0367 (15)	0.086 (2)	−0.0014 (11)	0.0084 (14)	0.0291 (15)
O3	0.090 (3)	0.112 (3)	0.041 (2)	0.009 (2)	0.0057 (19)	0.018 (2)
O4	0.054 (2)	0.101 (3)	0.093 (3)	−0.003 (2)	−0.031 (2)	−0.015 (2)
O5	0.0312 (14)	0.0391 (15)	0.0670 (19)	0.0026 (11)	0.0095 (13)	0.0276 (13)
O6	0.0265 (14)	0.0419 (15)	0.0569 (18)	−0.0024 (11)	−0.0010 (12)	0.0243 (13)
O7	0.093 (3)	0.0350 (18)	0.096 (3)	0.0016 (18)	−0.016 (2)	−0.0170 (18)
O8	0.065 (2)	0.135 (4)	0.065 (2)	0.032 (2)	−0.0004 (19)	−0.044 (2)
N1	0.050 (2)	0.053 (2)	0.049 (2)	0.0084 (18)	−0.0069 (19)	−0.0121 (18)
N2	0.056 (2)	0.059 (2)	0.042 (2)	0.0120 (19)	−0.0094 (18)	−0.0205 (18)
C1	0.035 (2)	0.0286 (19)	0.049 (2)	0.0023 (15)	0.0027 (17)	0.0075 (16)
C2	0.0245 (19)	0.032 (2)	0.075 (3)	0.0038 (15)	0.0060 (19)	0.0145 (19)
C3	0.0283 (18)	0.0251 (17)	0.043 (2)	0.0057 (14)	0.0039 (16)	0.0032 (15)
C4	0.0321 (19)	0.0316 (19)	0.039 (2)	0.0009 (15)	0.0023 (16)	−0.0012 (15)
C5	0.054 (3)	0.042 (2)	0.081 (4)	−0.013 (2)	0.012 (3)	0.004 (2)
C6	0.096 (4)	0.039 (3)	0.070 (4)	0.002 (3)	0.033 (3)	0.018 (2)
C7	0.102 (4)	0.053 (3)	0.040 (3)	0.024 (3)	0.002 (3)	0.013 (2)
C8	0.058 (3)	0.041 (2)	0.053 (3)	0.008 (2)	−0.019 (2)	0.001 (2)
C9	0.0315 (19)	0.0311 (19)	0.038 (2)	0.0021 (15)	0.0025 (16)	0.0100 (16)
C10	0.029 (2)	0.051 (2)	0.055 (3)	0.0056 (17)	0.0051 (18)	0.022 (2)
C11	0.035 (2)	0.0276 (18)	0.035 (2)	0.0015 (14)	−0.0060 (16)	0.0048 (15)
C12	0.040 (2)	0.038 (2)	0.030 (2)	−0.0071 (16)	−0.0068 (16)	−0.0018 (15)
C13	0.071 (3)	0.081 (4)	0.029 (2)	−0.024 (3)	0.000 (2)	0.005 (2)
C14	0.111 (5)	0.052 (3)	0.063 (4)	−0.038 (3)	−0.021 (3)	0.027 (3)
C15	0.103 (5)	0.029 (2)	0.083 (4)	−0.002 (3)	−0.027 (3)	0.011 (2)
C16	0.061 (3)	0.037 (2)	0.054 (3)	0.013 (2)	−0.009 (2)	0.002 (2)

### Geometric parameters (Å, º)

**Table:**

Ag1—O1	2.167 (2)	C3—C4	1.379 (5)
Ag1—O5	2.221 (2)	C3—C8	1.388 (5)
Ag1—O2^i^	2.405 (3)	C4—C5	1.365 (5)
Ag1—Ag2	2.8200 (4)	C5—C6	1.354 (7)
Ag1—Ag1^ii^	3.1998 (7)	C5—H5	0.9300
Ag2—O6	2.174 (2)	C6—C7	1.351 (7)
Ag2—O2	2.219 (3)	C6—H6	0.9300
Ag2—O5^iii^	2.404 (2)	C7—C8	1.384 (7)
Ag2—Ag2^iv^	3.2140 (7)	C7—H7	0.9300
O1—C1	1.241 (4)	C8—H8	0.9300
O2—C1	1.259 (4)	C9—C10	1.518 (5)
O2—Ag1^iii^	2.405 (3)	C10—C11	1.503 (5)
O3—N1	1.211 (5)	C10—H10A	0.9700
O4—N1	1.215 (5)	C10—H10B	0.9700
O5—C9	1.260 (4)	C11—C12	1.380 (5)
O5—Ag2^i^	2.404 (2)	C11—C16	1.380 (5)
O6—C9	1.244 (4)	C12—C13	1.386 (6)
O7—N2	1.214 (5)	C13—C14	1.373 (8)
O8—N2	1.212 (5)	C13—H13	0.9300
N1—C4	1.476 (5)	C14—C15	1.348 (8)
N2—C12	1.451 (5)	C14—H14	0.9300
C1—C2	1.509 (5)	C15—C16	1.354 (7)
C2—C3	1.498 (5)	C15—H15	0.9300
C2—H2A	0.9700	C16—H16	0.9300
C2—H2B	0.9700		
			
O1—Ag1—O5	162.60 (9)	C4—C3—C2	126.1 (3)
O1—Ag1—O2^i^	119.49 (9)	C8—C3—C2	118.4 (4)
O5—Ag1—O2^i^	76.16 (9)	C5—C4—C3	122.4 (4)
O1—Ag1—Ag2	86.12 (7)	C5—C4—N1	116.5 (4)
O5—Ag1—Ag2	77.51 (6)	C3—C4—N1	121.0 (3)
O2^i^—Ag1—Ag2	153.31 (6)	C6—C5—C4	120.4 (4)
O1—Ag1—Ag1^ii^	123.20 (8)	C6—C5—H5	119.8
O5—Ag1—Ag1^ii^	61.69 (8)	C4—C5—H5	119.8
O2^i^—Ag1—Ag1^ii^	86.43 (8)	C7—C6—C5	119.8 (4)
Ag2—Ag1—Ag1^ii^	84.972 (15)	C7—C6—H6	120.1
O6—Ag2—O2	161.91 (10)	C5—C6—H6	120.1
O6—Ag2—O5^iii^	118.69 (9)	C6—C7—C8	119.7 (4)
O2—Ag2—O5^iii^	76.23 (9)	C6—C7—H7	120.2
O6—Ag2—Ag1	86.72 (6)	C8—C7—H7	120.2
O2—Ag2—Ag1	77.83 (6)	C7—C8—C3	122.1 (4)
O5^iii^—Ag2—Ag1	154.04 (6)	C7—C8—H8	119.0
O6—Ag2—Ag2^iv^	119.58 (8)	C3—C8—H8	119.0
O2—Ag2—Ag2^iv^	65.40 (9)	O6—C9—O5	124.5 (3)
O5^iii^—Ag2—Ag2^iv^	95.20 (7)	O6—C9—C10	120.8 (3)
Ag1—Ag2—Ag2^iv^	74.507 (13)	O5—C9—C10	114.7 (3)
C1—O1—Ag1	121.2 (2)	C11—C10—C9	117.4 (3)
C1—O2—Ag2	129.1 (2)	C11—C10—H10A	107.9
C1—O2—Ag1^iii^	126.9 (2)	C9—C10—H10A	107.9
Ag2—O2—Ag1^iii^	102.88 (10)	C11—C10—H10B	107.9
C9—O5—Ag1	129.8 (2)	C9—C10—H10B	108.0
C9—O5—Ag2^i^	127.3 (2)	H10A—C10—H10B	107.2
Ag1—O5—Ag2^i^	102.86 (9)	C12—C11—C16	115.8 (4)
C9—O6—Ag2	120.7 (2)	C12—C11—C10	125.7 (3)
O3—N1—O4	124.4 (4)	C16—C11—C10	118.5 (4)
O3—N1—C4	118.4 (4)	C11—C12—C13	122.5 (4)
O4—N1—C4	117.1 (4)	C11—C12—N2	120.7 (4)
O8—N2—O7	123.6 (4)	C13—C12—N2	116.7 (4)
O8—N2—C12	118.7 (4)	C14—C13—C12	118.3 (5)
O7—N2—C12	117.7 (4)	C14—C13—H13	120.8
O1—C1—O2	124.6 (3)	C12—C13—H13	120.8
O1—C1—C2	119.8 (3)	C15—C14—C13	120.2 (5)
O2—C1—C2	115.6 (3)	C15—C14—H14	119.9
C3—C2—C1	117.0 (3)	C13—C14—H14	119.9
C3—C2—H2A	108.0	C14—C15—C16	120.4 (5)
C1—C2—H2A	108.0	C14—C15—H15	119.8
C3—C2—H2B	108.0	C16—C15—H15	119.8
C1—C2—H2B	108.0	C15—C16—C11	122.5 (5)
H2A—C2—H2B	107.3	C15—C16—H16	118.7
C4—C3—C8	115.5 (3)	C11—C16—H16	118.7
			
Ag1—O1—C1—O2	−12.5 (6)	Ag2—O6—C9—O5	−11.1 (5)
Ag1—O1—C1—C2	169.0 (3)	Ag2—O6—C9—C10	170.6 (3)
Ag2—O2—C1—O1	5.5 (6)	Ag1—O5—C9—O6	9.8 (6)
Ag1^iii^—O2—C1—O1	−160.2 (3)	Ag2^i^—O5—C9—O6	−167.2 (3)
Ag2—O2—C1—C2	−175.9 (3)	Ag1—O5—C9—C10	−171.8 (3)
Ag1^iii^—O2—C1—C2	18.3 (5)	Ag2^i^—O5—C9—C10	11.2 (5)
O1—C1—C2—C3	−1.4 (6)	O6—C9—C10—C11	−8.0 (6)
O2—C1—C2—C3	−180.0 (4)	O5—C9—C10—C11	173.6 (4)
C1—C2—C3—C4	−57.6 (6)	C9—C10—C11—C12	−47.7 (6)
C1—C2—C3—C8	122.1 (4)	C9—C10—C11—C16	131.7 (4)
C8—C3—C4—C5	−1.7 (6)	C16—C11—C12—C13	−3.2 (6)
C2—C3—C4—C5	177.9 (4)	C10—C11—C12—C13	176.2 (4)
C8—C3—C4—N1	176.2 (3)	C16—C11—C12—N2	174.5 (3)
C2—C3—C4—N1	−4.1 (6)	C10—C11—C12—N2	−6.1 (6)
O3—N1—C4—C5	155.5 (4)	O8—N2—C12—C11	150.3 (4)
O4—N1—C4—C5	−23.1 (5)	O7—N2—C12—C11	−31.5 (5)
O3—N1—C4—C3	−22.5 (6)	O8—N2—C12—C13	−31.9 (5)
O4—N1—C4—C3	158.8 (4)	O7—N2—C12—C13	146.4 (4)
C3—C4—C5—C6	2.5 (7)	C11—C12—C13—C14	2.7 (6)
N1—C4—C5—C6	−175.5 (4)	N2—C12—C13—C14	−175.1 (4)
C4—C5—C6—C7	−1.0 (8)	C12—C13—C14—C15	0.7 (7)
C5—C6—C7—C8	−1.0 (8)	C13—C14—C15—C16	−3.4 (8)
C6—C7—C8—C3	1.8 (7)	C14—C15—C16—C11	2.8 (8)
C4—C3—C8—C7	−0.4 (6)	C12—C11—C16—C15	0.4 (6)
C2—C3—C8—C7	179.9 (4)	C10—C11—C16—C15	−179.0 (4)

Symmetry codes: (i) *x*+1, *y*, *z*; (ii) −*x*+2, −*y*, −*z*; (iii) *x*−1, *y*, *z*; (iv) −*x*+1, −*y*, −*z*.

### Hydrogen-bond geometry (Å, º)

**Table:**

*D*—H···*A*	*D*—H	H···*A*	*D*···*A*	*D*—H···*A*
C2—H2*B*···O4^iii^	0.97	2.53	3.312 (6)	138
C10—H10*B*···O8^i^	0.97	2.35	3.205 (6)	146
C6—H6···O8^v^	0.93	2.48	3.322 (6)	151
C15—H15···O7^vi^	0.93	2.59	3.268 (6)	130

Symmetry codes: (i) *x*+1, *y*, *z*; (iii) *x*−1, *y*, *z*; (v) *x*, −*y*+1/2, *z*−1/2; (vi) −*x*+2, *y*−1/2, −*z*+1/2.
