# Supplementary figures and images for: Crystal structure of catena-poly[bis­[μ3-2-(2-nitro­phen­yl)acetato-κ3 O:O:O′]disilver(I)]
Source: Acta Crystallogr E Crystallogr Commun. 2015 Apr 25;71(Pt 5):m118–9. doi: 10.1107/S2056989015007616 (PMC4420127; doi:10.1107/S2056989015007616)

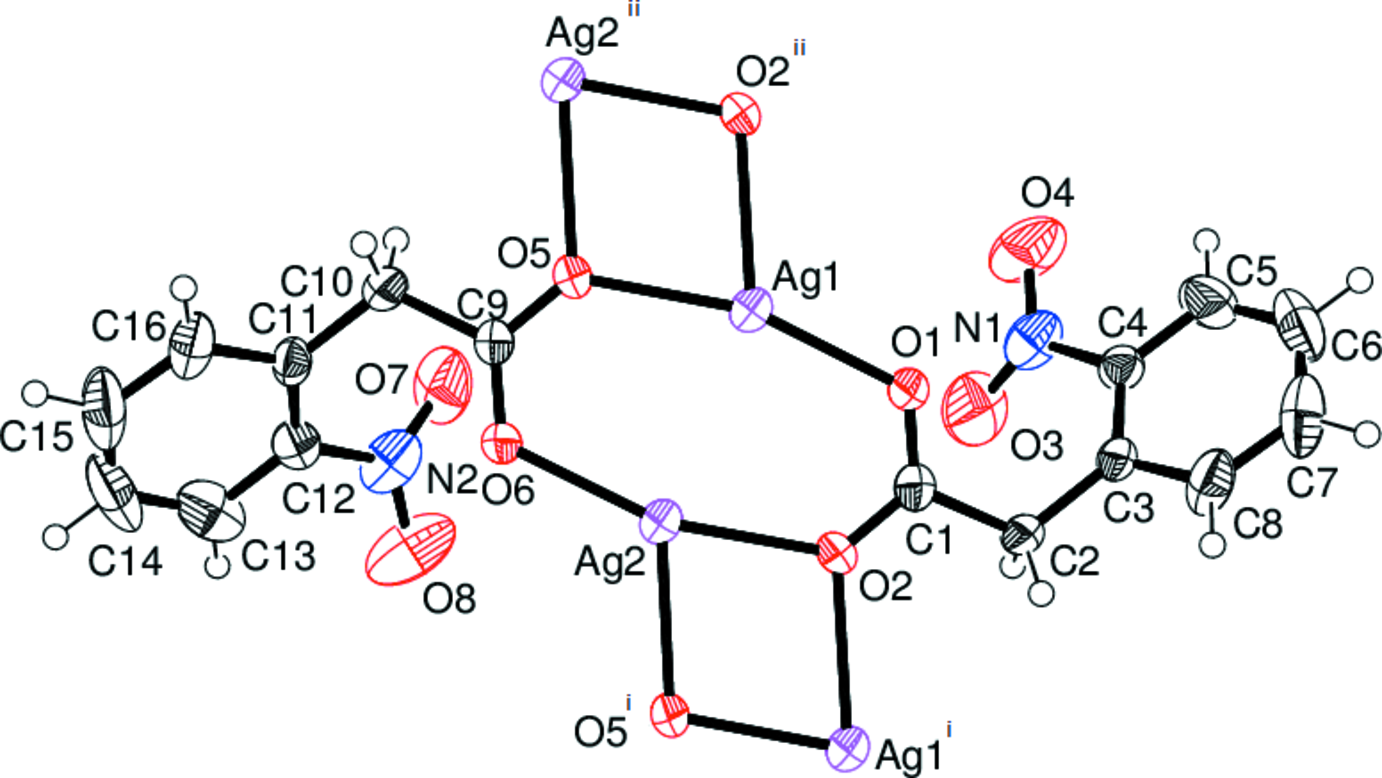

Supplement: Supplementary file 3 [file e-71-0m118-fig1.tif]

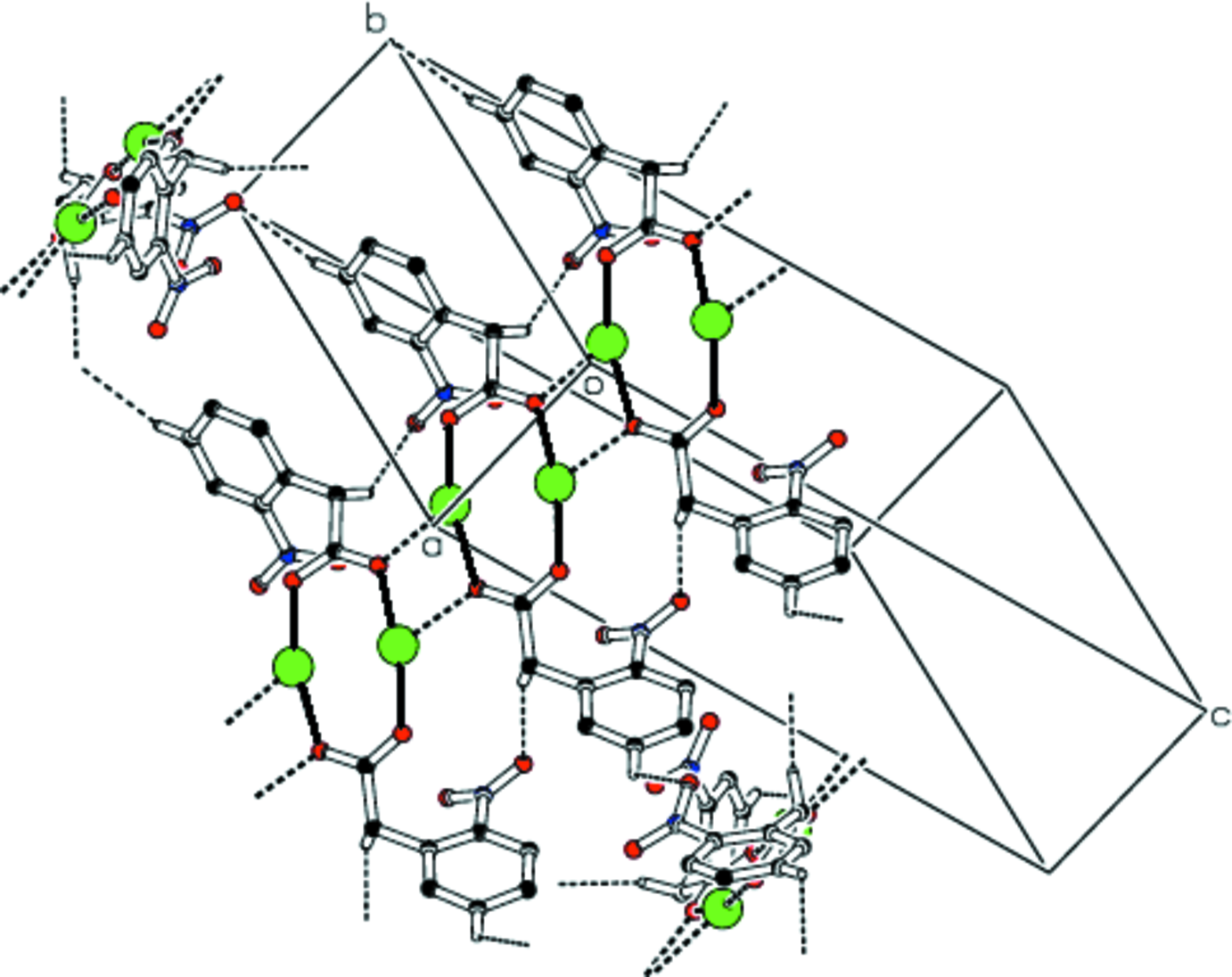

Supplement: Supplementary file 4 [file e-71-0m118-fig2.tif]
